# Fragile X mental retardation protein: from autism to neurodegenerative disease

**DOI:** 10.3389/fncel.2015.00043

**Published:** 2015-02-12

**Authors:** Hansen Wang

**Affiliations:** Faculty of Medicine, University of TorontoToronto, ON, Canada

**Keywords:** FMRP, Alzheimer disease, fragile X syndrome, autism, β amyloid, neurodegeneration, tau, synaptic plasticity

## Introduction

Fragile X mental retardation protein (FMRP) is a RNA binding protein, the absence of which due to silencing of the *FMR1* gene causes fragile X syndrome, an X-linked neurodevelopmental disorder (Bassell and Warren, 2008; Bhakar et al., 2012; Santoro et al., 2012). FMRP regulates the transport, stability and translation of its mRNA targets. Loss of FMRP alters translational control and receptor-mediated signaling pathways, leading to aberrant dendritic spine dynamics, synaptic dysfunction and behavioral phenotypes in fragile X syndrome (Wang et al., 2012; Sidorov et al., 2013; Suhl et al., 2014). Since the identification of FMR1 as the causative gene for fragile X syndrome in 1991, studies have mainly focused on understanding the function of FMRP. More and more potential FMRP mRNA targets and interacting proteins have been identified in the mammalian brain, supporting the critical roles of FMRP in neurodevelopment, synaptic plasticity and other neurological disorders apart from fragile X syndrome (Wang et al., 2008b, 2012; Pasciuto and Bagni, 2014a; Suhl et al., 2014).

Fragile X syndrome, the most common monogenic cause of autism spectrum disorders (ASDs), has been leading the way for better understanding of autism and other neurodevelopmental disorders (Belmonte and Bourgeron, 2006; Bhakar et al., 2012; Banerjee et al., 2014; Cook et al., 2014). Clinically, a substantial proportion of children with fragile X syndrome meets diagnostic criteria for ASDs (Budimirovic and Kaufmann, 2011). Genetically and biologically, many of the neuronal targets of FMRP overlap with genes associated with ASDs, suggesting the common pathways that are dysregulated and might be potential therapeutic targets for these neurodevelopmental disorders (Auerbach et al., 2011; Zoghbi and Bear, 2012; Darnell and Klann, 2013). Interestingly, studies in recent years have further revealed that FMRP regulates a multitude of synaptic proteins and components of signaling pathways that not only affect neurodevelopment, but also contribute to the pathogenesis of neurodegenerative diseases such as Alzheimer disease (AD), the leading cause for dementia in the elderly (Malter et al., 2010; Sokol et al., 2011; Westmark et al., 2011; Hamilton et al., 2014). FMRP may play a pivotal role in the association between neurodevelopmental and neurodegenerative disorders across lifespan.

## FMRP and AD pathogenesis

AD is pathologically characterized by the presence of plaques comprised of β amyloid (Aβ) and neurofibrillary tangles (NFTs) containing hyperphosphorylated tau protein (Selkoe, 2011; Holtzman et al., 2012; Ubhi and Masliah, 2013; Bloom, 2014). A considerable amount of evidence suggests that soluble Aβ oligomers are the predominant neurotoxic species of Aβ, with Aβ 42 fragment as the particularly potent form (Klyubin et al., 2012; Masters and Selkoe, 2012; Klein, 2013). Aβ oligomers exert the toxic effects by binding to their receptors on neuronal synapses, disrupting normal synaptic signaling pathways, which further leads to synaptic damage accompanied by neuronal loss (Benilova et al., 2012; Sheng et al., 2012; Pozueta et al., 2013; Wang et al., 2013; Tu et al., 2014).

### FMRP in Aβ mediated synaptic toxicity

**Figure 1:**
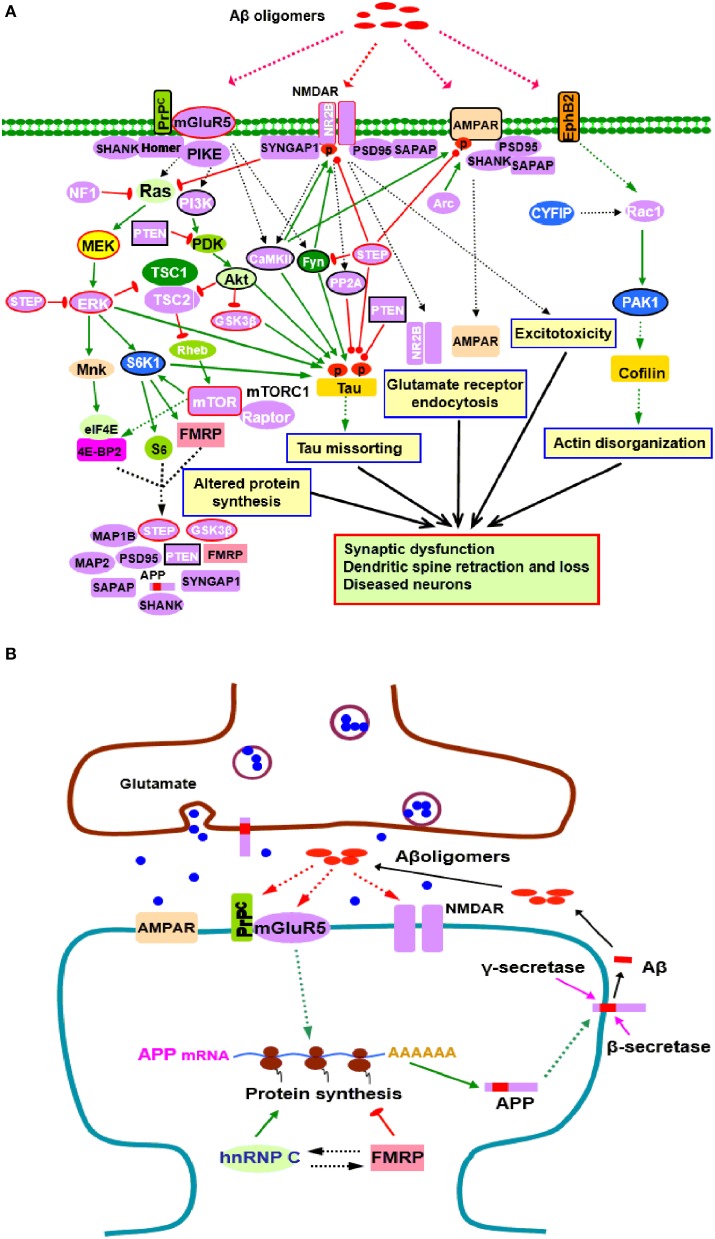
**Potential roles of FMRP in the pathogenesis of AD**. **(A)** FMRP might be involved in oligomeric Aβ induced neurotoxicity. At pathological concentrations, Aβ oligomers may interact with multiple neuronal synaptic receptors such as mGluR5-PrP^C^, NMDARs, AMPARs, and EphB2, triggering a series of toxic synaptic events which may involve FMRP and eventually lead to synaptic dysfunction and neuronal loss. These events include: Aberrant activation of PI3K-Akt-mTORC1 and MEK-ERK signaling pathways linked to cap-dependent translation result in altered synthesis of synaptic proteins; Oligomeric Aβ exposure disrupts the balance between tau kinase (GSK3β, CaMKII, Akt, Fyn, and ERK1/2) and phosphatase (PP2A, STEP, and PTEN) activities, inducing tau hyperphosphorylation and aggregation; Stimulating EphB2-Rac1/PAK1 signaling by Aβ oligomers induces cofilin phosphorylation and actin depolymerization, leading to actin network disorganization; Binding of Aβ oligomers to PrP^C^-mGluR5 activates Fyn kinase which phosphorylates not only tau, but also NR2B subunit of NMDARs, enhancing NMDAR activity and causing excitotoxicity; STEP is also activated, inactivates Fyn, and dephosphorylates AMPARs and NMDARs, resulting in endocytosis of glutamate receptors, a cellular process involves Arc, PSD-95, SAPAP, and other synaptic proteins. Purple proteins are those whose mRNAs are FMRP targets (Darnell and Klann, 2013; Pasciuto and Bagni, 2014b; Santini and Klann, 2014); the blue ones are the interacting proteins of FMRP (Pasciuto and Bagni, 2014a). Proteins with red lines around them indicate those that have been successfully manipulated either pharmacologically or genetically to reverse molecular, cellular and/or behavioral phenotypes in animal models of AD (Zhang et al., 2010; Malinow, 2012; Caccamo et al., 2014; Feld et al., 2014; Hamilton et al., 2014; Llorens-Martin et al., 2014) as well as ASDs (Goebel-Goody et al., 2012; Guo et al., 2012; Won et al., 2012; Darnell and Klann, 2013; Osterweil et al., 2013; Wang and Doering, 2013; Wang, 2014). Proteins with black lines around them are the ones that have been reported to be potential targets for AD therapy (Griffin et al., 2005; Lafay-Chebassier et al., 2005; Ma et al., 2008; Cisse et al., 2011; Moriguchi, 2011; Chang et al., 2012; Gross and Bassell, 2014; Nygaard et al., 2014; Sontag and Sontag, 2014). **(B)** FMRP in APP synthesis. Aβ oligomers stimulate dendritic APP synthesis through PrP^C^-mGluR5 mediated protein translation dependent pathway, providing template for secretase cleavage to produce Aβ and other metabolites. A positive feedback may exist whereby production of APP results in increased substrate for amyloidogenic processing and release of Aβ, which then acitivates mGluR5 to further stimulate APP translation. In this process, FMRP competes with the other RNA binding protein hnRNP C to modulate APP translation. FMRP is a repressor of APP translation, whereas hnRNP C acts as an enhancer. The rate of APP synthesis is directly influenced by the relative association of each RNA binding protein (Lee et al., 2010). In signaling pathways, arrows indicate positive (green) or inhibitory (red) consequence on downstream components, but they do not necessarily represent direct interactions.

A growing number of synaptic proteins have been proposed as potential Aβ receptors or coreceptors, which are believed to mediate Aβ induced synaptic dysfunction (Karran et al., 2011; Paula-Lima et al., 2013; Pozueta et al., 2013; Overk and Masliah, 2014). Those receptors include, but are not limited to, NMDARs, mGluR5, AMPARs, cellular prion protein (PrP^C^), PSD-95, and EphB2 (Lacor et al., 2004; Lauren et al., 2009; Cisse et al., 2011; Larson and Lesne, 2012; Mucke and Selkoe, 2012; Um et al., 2013; Tu et al., 2014). In fact, some of Aβ receptors (NMDARs, mGluR5, and PSD-95) and their associated scaffolding proteins and adhesion molecules such as SAPAP, Shank, Homer, and SynGAP1, are those whose mRNAs are FMRP targets (Darnell and Klann, 2013; Santini and Klann, 2014), suggesting that FMRP might be involved in initiating toxic effects of Aβ oligomers through regulating Aβ receptors (Figure 1A).

Aβ induced synaptic dysfunction/loss is a complicated process involving multiple signaling pathways and biological events (Benilova et al., 2012; Tu et al., 2014). FMRP may be implicated in this process through selective regulation of components in those signaling pathways perturbed by Aβ oligomers (Figure 1A). Binding of glutamate receptors (NMDARs, mGluR5, and AMPARs) by Aβ oligomers impairs glutamatergic neurotransmission and triggers aberrant activation of downstream pathways, including PI3K-Akt-mTOR and MEK-ERK signaling pathways (Benilova et al., 2012; Pozueta et al., 2013) which are coupled to cap-dependent translation (Darnell and Klann, 2013; Santini and Klann, 2014). FMRP may regulate the activity of these translational control pathways directly since several components (PIKE, PI3K, mTOR, S6K1, and ERK) and negative regulators (PTEN, NF1, and STEP) of the pathways are FMRP targets (Figure 1A). FMRP also plays a critical role in regulating the balance of cap-dependent translation of its target mRNAs (Darnell and Klann, 2013; Sidorov et al., 2013; Santini and Klann, 2014). Dysregulation of the translational control pathways caused by Aβ oligomers will affect the protein products of FMRP targets, including Aβ precursor protein (APP), ARC, STEP, PTEN, GSK3β and other molecules which are closely linked to AD or other neurodegenerative disorders (Figure 1A), further associating FMRP with neurodegeneration.

Aβ induced synaptic degeneration also involves surface removal and endocytosis of glutmate receptors (NMDARs and AMPARs) (Um et al., 2012; Boehm, 2013; Tu et al., 2014). The FMRP targets, such as the scaffolding protein PSD-95 and SAPAP3, immediate-early gene product Arc, protein kinase CaMKII and tyrosine phosphatase STEP, are key determinants of NMDAR or AMPAR endocytosis. PSD-95 and SAPAP3 tethers the receptors to signaling proteins and neuronal cytoskeleton (Wang et al., 2010; Chen et al., 2011); Arc is a component of AMPAR endocytosis machinery and regulates the endocytosis rate (Chowdhury et al., 2006; Waung et al., 2008); CaMKII and STEP mediate surface expression or internalization of NMDARs and AMPARs through phosphorylation/dephosphorylation of relative receptor subunits (Wang et al., 2008b; Zhang et al., 2008; Lisman et al., 2012), supporting possible involvement of FMRP in glutmate receptor endocytosis (Figure 1A). The actin disorganization contributes to AD pathogenesis (Maloney and Bamburg, 2007; Bamburg and Bloom, 2009; Penzes and Vanleeuwen, 2011). The Rac1/PAK1 signaling downstream of EphB2 is involved in actin cytokeleton remodeling; CYFIP1/2 are linked to Rac1. Binding of EphB2 by Aβ oligomers stimulates PAK1, which further induces cofilin phosphorylation to mediate actin depolymerization, thereby inducing actin network disorganization, dendritic spine shrinkage and loss (Ma et al., 2012; Abekhoukh and Bardoni, 2014; Cisse and Checler, 2014). In this signaling pathway, Rac1 is a FMRP target, and CYFIP1/2 and PAK interact with FMRP (Figure 1A). Thus, FMRP is likely to play a role in actin disorganization in AD pathogenesis.

The presence of hyperphosphorylated tau enriched NFTs is a classical AD pathological hallmark. Tau, a microtubule associated protein (MAP), becomes hyperphosphorylated and disassociated from microtubules under pathological conditions, subsequently forming soluble aggregates, insoluble filaments, and eventually NFTs in affected brain regions (Medina and Avila, 2014; Zempel and Mandelkow, 2014). Studies have shown that Aβ-induced synaptic loss and toxicity are tau dependent (Vossel et al., 2010; Roberson et al., 2011). There is a causal association between oligomeric Aβ exposure and tau phosphorylation (Larson et al., 2012; Boehm, 2013). Tau phosphorylation is regulated by a balance between tau kinase and phosphatase activities. Disruption of this balance by Aβ exposure is suggested to cause abnormal tau phosphorylation and thereby contributes to tau aggregation (Wang et al., 2007; Martin et al., 2013a,b). Of the tau protein kinases, GSK3β, CaMKII, ERK1/2, and S6K1 are known to be FMRP targets (Figure 1A). The decrease in the levels or activity of protein phosphatase(s) that dephosphorylate tau also contributes to AD pathology (Braithwaite et al., 2012; Martin et al., 2013a); among these phosphatases, PP2A, STEP, and PTEN are FMRP targets (Figure 1A). FMRP thus, might be involved in tau pathology through regulating those tau kinases and phosphatases. Hyperphosphorylated tau sequesters normal tau, and the other two major MAPs (MAP1 and MAP2), causing disruption of microtubules and misfolding of tau (Zempel and Mandelkow, 2014). Notably, both MAP1A and MAP2 are FMRP targets, further implicating FMRP in neurofibrillary degeneration (Figure 1A).

### FMRP in Aβ stimuiated APP synthesis

Aβ is produced by the sequential proteolytic cleavage of APP by β - and γ-secretases via amyloidogenic pathway (Masters and Selkoe, 2012). The expression of APP can be upregulated upon mGluR5 stimulation. FMRP, which is also regulated by mGluR5 (Ronesi and Huber, 2008; Wang et al., 2008a; Wang and Zhuo, 2012), binds to and represses the translation of APP mRNA due to mGluR5 activation (Westmark and Malter, 2007; Westmark, 2013).

The mGluR5 links FMRP with APP. It is known that mGluR5 acts as a coreceptor for Aβ oligomers bound to PrP^C^ (Um et al., 2013). Aβ oligomers can stimulate APP synthesis through the mGluR5 and protein translation dependent pathway which involves FMRP, providing template for secretase cleavage to produce Aβ and other metabolites (Westmark and Malter, 2007; Westmark, 2013). Aβ oligomer interactions with mGluR5-PrP^C^ may function to accelerate Aβ production through the FMRP dependent signaling pathway, suggesting that a positive feedback loop may exist in AD, whereby translation of APP results in increased substrate for amyloidogenic processing and generation of Aβ which then stimulates mGluR5 signaling to induce further synaptic synthesis of APP (Westmark and Malter, 2007; Westmark, 2013) (Figure 1B). Thus, in addition to the established role in fragile X syndrome and autism, FMRP likely contributes directly to AD pathogenesis through mGluR5 dependent APP production.

## FMRP targets and AD therapy

As discussed above, a number of signaling pathways, including PI3K-Akt-mTORC1, MEK-ERK and PAK1 pathways, have been found to be involved in the neurodegenerative progression of AD. Therapies for AD might require the development of drugs targeting these aberrant signaling pathways, among which several key signaling proteins such as PI3K, mTOR, ERK and PAK1, are targets of FMRP. In addition, the FMRP targeted Aβ oligomer receptors including mGluR5 and NMDARs could be ideal therapeutic targets for AD (Figure 1A). Particularly, pharmacological inhibition or genetic deletion of mGluR5 was recently found to rescue learning deficits, or reduce Aβ oligomers and plaques in AD mice (Um et al., 2013; Hamilton et al., 2014).

Tau plays crucial roles in the neuronal cytoskeleton stabilization and is an important target for AD therapies (Gotz et al., 2012; Himmelstein et al., 2012; Giacobini and Gold, 2013). Interventions focused on preventing or reducing tau hyperphosphorylation and mislocalization may provide additional strategies for treatment of AD. The therapeutic tactics include Tau kinase inhibitors and phosphatase activators (Giacobini and Gold, 2013; Zempel and Mandelkow, 2014). The relevant kinases and phosphatases could be the FMRP targets such as GSK3β, ERK, S6K1, PP2A, PTEN, and STEP (Figure 1A). Although the tau based treatments are encouraging, additional work are undoubtedly needed to optimize each treatment for further development of safe and effective therapies.

Therefore, FMRP targeted signaling molecules not only provide therapeutic strategies for fragile X syndrome and other ASDs (Darnell and Klann, 2013; Santini and Klann, 2014), but may serve as potential targets for treatment of AD. Indeed, many components of altered signaling pathways in AD overlap with those in ASDs. A number of signaling proteins targeted by FMRP have been successfully manipulated either pharmacologically or genetically to reverse molecular, cellular and/or behavioral phenotypes in animal models of both ASDs and AD (Figure 1A). FMRP thus, acts as a molecular link between ASDs and AD through the common signaling pathways among the diseases. Developing novel therapies directed at FMRP targets may benefit both neurodevelopmental and neurodegenerative disorders.

## Future perspectives

It is now known that FMRP controls signaling pathways that could be associated with both neurodevelopmental and neurodegenerative disorders. FMRP not only regulates gene expression at the translational level, but also interacts with a multitude of proteins at both presynaptic and postsynaptic sites (Pasciuto and Bagni, 2014a; Myrick et al., 2015). However, so far no systemic proteomic analysis of FMRP interactome in brain has been reported. Additionally, although many of the interacting proteins of FMRP such as transactive response DNA-binding protein-43 (TDP-43), survival of motor neuron 1 (SMN1) and CYFIP are known to be linked to neurological disorders (Abekhoukh and Bardoni, 2014; Pasciuto and Bagni, 2014a), the significance of the protein-protein interaction to individual proteins and diseases still need to be further characterized.

The AD animal or cell models are powerful tools for investigating the pathogenesis of the neurodegenerative disease. It will be useful to set up the transgenic AD mice/Aβ-treated primary neuronal culture which either lack or overexpress FMRP for further evaluating the pathological role of FMRP in AD. FMRP regulates specific mRNA/protein targets at different developmental stages and in different brain areas. Future studies will need to provide detailed information on FMRP mRNA targets and FMRP interactome in relevant brain areas at specific developmental stages of AD animal models. The information will greatly help to further elucidate the pathogenesis of this neurodegenerative disease and develop relative therapeutic strategies.

### Conflict of interest statement

The author declares that the research was conducted in the absence of any commercial or financial relationships that could be construed as a potential conflict of interest.

## Abbreviations

4E-BP2: eIF4E-binding protein 2
AD: Alzheimer disease
Aβ: β amyloid
AMPAR: α-amino-3-hydroxyl-4-isoxazole propionic acid receptors
APP: amyloid precursor protein
Arc: activity-regulated cytoskeleton-associated protein
CYFIP: cytoplasmic FMRP interacting protein
eIF4E: eukaryotic initiation factor 4E
EphB2: ephrin type-B receptor 2
ERK: extracellular signal related kinase
FMRP: fragile X mental retardation protein
GABA: gamma aminobutyric acid
GSK3β: glycogen synthase kinase-3β
hnRNP C: heterogeneous nuclear ribonucleoprotein C
MAP1A/2: microtubule associated protein 1A/2
MEK: mitogen-activated protein kinase/ERK kinase
mGluR5: metabotropic glutamate receptor 5
Mnk: mitogen-activated protein kinase interacting kinase
mTOR: mammalian target of rapamycin
mTORC1: mammalian target of rapamycin complex 1
NF1: neurofibromatosis 1
NMDAR: N-methyl-d-aspartate receptors
PAK1: p21-activated kinase 1
PI3K: phosphatidylinositol 3-kinase
PIKE: PI3K enhancer
PP2A: protein phosphatase 2A
PrP^C^: cellular prion protein
PSD-95: postsynaptic density 95
PTEN: Phosphatase and tensin homolog
Raptor: regulatory-associated protein of mTOR
S6K1: p70 ribosomal kinase 1
SAPAP: SAP90/PSD-95–associated protein
SHANK: Src homology 3 (SH3) and multiple ankyrin repeat domains protein
STEP: striatal-enriched protein tyrosine phosphatase
SYNGAP1: synaptic Ras guanosine triphosphatase (GTPase)–activating protein 1
TSC1/2: tuberous sclerosis complex 1/2.
